# Genome-enabled predictions for fruit weight and quality from repeated records in European peach progenies

**DOI:** 10.1186/s12864-017-3781-8

**Published:** 2017-06-06

**Authors:** Filippo Biscarini, Nelson Nazzicari, Marco Bink, Pere Arús, Maria José Aranzana, Ignazio Verde, Sabrina Micali, Thierry Pascal, Benedicte Quilot-Turion, Patrick Lambert, Cassia da Silva Linge, Igor Pacheco, Daniele Bassi, Alessandra Stella, Laura Rossini

**Affiliations:** 1 PTP Science Park, Via Einstein - Loc. Cascina Codazza, Lodi, Italy; 2 Council for Agricultural Research and Economics (CREA) Research Centre for Fodder Crops and Dairy Productions, Lodi, Italy; 3 Wageningen UR Biometris, Wageningen, The Netherlands; 4 IRTA, Centre de Recerca en Agrigenòmica CSIC-IRTA-UAB-UB, Campus UAB, Bellaterra (Cerdanyola del Vallés), Barcelona, Spain; 5 Consiglio per la ricerca in agricoltura e l’analisi dell’economia agraria (CREA) — Centro di Ricerca per la Frutticoltura (CREA-FRU), Via di Fioranello 52, Roma, Italy; 6 GAFL, INRA, Montfavet, 84140 France; 7 Università degli Studi di Milano - DiSAA, Via Celoria 2, Milano, Italy; 8 IBBA-CNR, Via Edoardo Bassini, 15, Milan, 20133 Italy; 9 Institute of Nutrition and Food Technology - INTA, Universidad de Chile, Av El Líbano 5524, Santiago, Chile; 10 Present Address: Hendrix Genetics Research, Technology & Services B.V., P.O. Box 114, Boxmeer NL, 5830AC The Netherlands

**Keywords:** Peach (*Prunus persica*), Genome-enabled predictions, Fruit weight, Sugar content, Titratable acidity, Genotype imputation, Repeatability model

## Abstract

**Background:**

Highly polygenic traits such as fruit weight, sugar content and acidity strongly influence the agroeconomic value of peach varieties. Genomic Selection (GS) can accelerate peach yield and quality gain if predictions show higher levels of accuracy compared to phenotypic selection. The available IPSC 9K SNP array V1 allows standardized and highly reliable genotyping, preparing the ground for GS in peach.

**Results:**

A repeatability model (multiple records per individual plant) for genome-enabled predictions in eleven European peach populations is presented. The analysis included 1147 individuals derived from both commercial and non-commercial peach or peach-related accessions. Considered traits were average fruit weight (FW), sugar content (SC) and titratable acidity (TA). Plants were genotyped with the *9K IPSC* array, grown in three countries (France, Italy, Spain) and phenotyped for 3–5 years. An analysis of imputation accuracy of missing genotypic data was conducted using the software Beagle, showing that two of the eleven populations were highly sensitive to increasing levels of missing data. The regression model produced, for each trait and each population, estimates of heritability (FW:0.35, SC:0.48, TA:0.53, on average) and repeatability (FW:0.56, SC:0.63, TA:0.62, on average). Predictive ability was estimated in a five-fold cross validation scheme within population as the correlation of true and predicted phenotypes. Results differed by populations and traits, but predictive abilities were in general high (FW:0.60, SC:0.72, TA:0.65, on average).

**Conclusions:**

This study assessed the feasibility of Genomic Selection in peach for highly polygenic traits linked to yield and fruit quality. The accuracy of imputing missing genotypes was as high as 96%, and the genomic predictive ability was on average 0.65, but could be as high as 0.84 for fruit weight or 0.83 for titratable acidity. The estimated repeatability may prove very useful in the management of the typical long cycles involved in peach productions. All together, these results are very promising for the application of genomic selection to peach breeding programmes.

**Electronic supplementary material:**

The online version of this article (doi:10.1186/s12864-017-3781-8) contains supplementary material, which is available to authorized users.

## Background

Peach (*Prunus persica* L. Batsch) has been bred and cultivated for more than 4 000 years [1] and is both an important crop and a model species for the Rosaceae family [2]. The total world peach production was 21.6 million tonnes in 2014, of which 18.5*%* (4 million tonnes) from Europe [3]. Like most fruit trees, peach is a perennial crop. Because of their long juvenile phase, breeding perennial plants is a complex task that requires careful planning and precise economic evaluations [4]. In addition, varieties need to be tested in multiple locations over multiple years to assess their adaptation to the geographical environment and their production potential. The generation interval in current peach breeding programmes can be up to 5–7 years [5], and this limits the genetic gain potentially achievable per unit of time. An additional complication is that several relevant phenotypes are typically measured late in life (e.g. fruit size, plant yield, maturation time), thereby increasing the length and costs (e.g. keeping selection candidates) of peach breeding programmes.

In species with long breeding cycles, genomic selection bears the potential of improving selection efficiency –through e.g. reduced generation intervals, thereby speeding up genetic progress [6]. This was the major motivation behind the swift uptake of genomic selection in dairy cattle [7]. The relative high economic value of cattle, helped dairy cattle breeders pioneer the use of genomics in agriculture [8]. The constant decrease of sequencing costs and the availability of SNP genotyping technologies for an ever increasing number of species [9–11], has expanded the interest for genomic selection in modern breeding programmes. The genome sequence of peach is available [12], and an updated version has been recently released [13]. SNP chips for *P. persica* have also been designed [14].

The availability of SNP data allows –given a reference population that is both genotyped and phenotyped– for genomic predictions of unobserved phenotypes and genetic values for relevant traits in selection candidates, which is an essential element for the application of genomic selection to breeding. Genomic predictions for a variety of traits have been successfully modelled in a wide range of plant species e.g. the forage crop alfalfa [15], sugar beet [16, 17], loblolly pine [18], eucalyptus [19], including some important fruit trees like apple [20] and pear [21]. Traits considered focussed initially on yield and fruit size, but interest is growing also for traits related to fruit quality and response to environmental conditions, for life cycle traits (longevity, disease resistance, adaptability etc.), and for multiple-trait selection [22].

In perennial plants, the long life cycles and multiple records over successive years call for the modelling of repeated records. Not only the genetic/breeding value of plants is relevant for selection, but also the possibility of predicting the future performance, for management purposes. Repeatability models have found widespread application in animal breeding [23], while their use in plant breeding has been limited (e.g. maize [24]; cashew [25]).

In this paper, a repeatability model for genome-enabled predictions in eleven European peach populations is presented, where repetition refers to measurements in multiple successive years. Traits considered were fruit weight, and sugar content and acidity, which are key traits related to the quality of the fruit. To our knowledge, this is the first time that genomic predictions for any traits are reported in *P. persica*, and the first application overall of a repeatability model to genomic predictions in plants. The heritability, repeatability and predictive ability for the three phenotypic traits in each peach population are reported. In addition, the accuracy of missing genotypes imputation has been estimated, and statistical issues related to genome-enabled predictions have been discussed.

## Methods

### Plant material and genotypes

From research fields in Italy, France and Spain, 1 147 peach plants from 11 crosses were available: four crosses from Italy (459 plants), two crosses from France (250 plants), and five crosses from Spain (438 plants). Italian crosses came from orchards of the University of Milan and of the Fruit Tree Research Centre (CREA-FRU) in Roma; French crosses from orchards at INRA-Avignon; Spanish crosses from orchards at IRTA in Lleida. The crosses were: Bolero x Oro (BxO), Max x Rebus028 (MxR028), PI91459 (NJ Weeping) x Bounty (WxBy), and IF7310828 x (IF7310828 x Ferganensis) (PxF) from Italy; Bolinha x Bolinha (BoxBo) and (SD40 x Summergrand) x Zéphyr (BC2) from France; Big Top x Armking (BtxAk), Belbinette x Nectalady (BbxNl), Big Top x Nectacross (BtxNr), MB1.73 x Earlygold (T1E) and MB1.73 x MB1.73 (TxE) from Spain. The parental “SD40” originated from a cross between *P. persica* and *P. davidiana*.

All plants were genotyped with the peach IPSC 9K SNP array [14, 26], with an average call-rate of 96.7*%*. Of the initial 1 147 samples, 57 had a call-rate ≤0.90 and were discarded. Of the initial 8 144 SNP markers, 2 068 SNP that were monomorphic in all populations or had a call-rate ≤0.90 were removed from the dataset, leaving 6 076 SNP for the analysis. The residual average missing rate was 3.19*%* (1.17*%* in BbxNl; 5.79*%* in TxE). A summary of plant populations (i.e. progenies) and genotype data can be found in Table 1.

**Table 1:** Distribution of plants per cross and summary of SNP-genotype data

Cross	Code	Origin	Samples	CR >0.9	MR	MAF=0	Mean MAF
Belbinette x Nectalady	BbxNl	Spain	102	102	0.01	48.90%	28.30%
Big Top x Armking	BtxAk	Spain	77	74	0.01	45.50%	44.10%
Big Top x Nectacross	BtxNr	Spain	51	43	0.03	49.00%	47.60%
Bolinha x Bolinha	BoxBo	France	115	111	0.03	42.20%	40.40%
Bolero x OroA	BxO	Italy	129	126	0.02	37.50%	34.60%
IF7310828 x (IF7310828 x Ferganensis)	PxF	Italy	130	130	0.04	22.10%	22.60%
Max10 x Rebus	MxR028	Italy	73	72	0.04	49.50%	32.70%
MB1.73 x Earlygold	T1E	Spain	148	124	0.04	9.40%	21.10%
MB1.73 x MB1.73	TxE	Spain	60	54	0.06	30.40%	27.50%
(SD40 x Summergrand) x Zéphyr	BC2	France	135	131	0.04	19.00%	24.00%
NJ Weeping x Bounty	WxBy	Italy	127	123	0.03	40.50%	36.20%
Entire dataset			1147	1090	0.03	2.20%	26.40%

*CR*>0.9: call-rate >0.90; MR: residual missing rate after editing on the whole dataset; *MAF*=0: monomorphic SNP

### Phenotypic data

Fruit weight, sugar content and acidity measurements were available for the 1 090 peach trees left after editing for call-rate from the 11 *P. persica* populations. Fruit weight (FW) in grams was measured as the average weight of 10 random peaches sampled from each tree. Sugar content (SC) and titratable acidity (TA) were measured, respectively, as average Brix degrees (soluble solid content) and meq/100 ml in the juice of at least five ripe fruits. FW was available for all 11 peach crosses; SC and TA only for 9 crosses (all except BoxBo and WxBy).

**Fig. 1:**
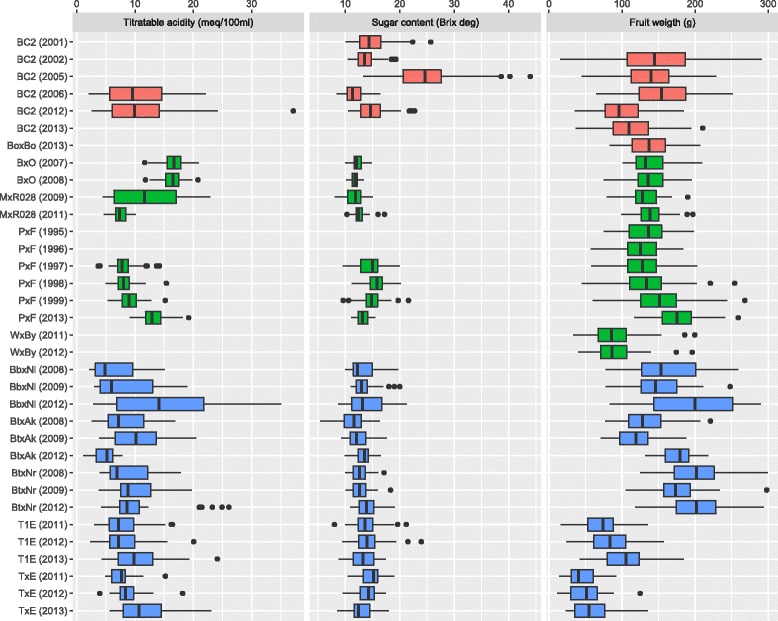
Boxplots of phenotypic records per trait, year and cross. *Crosses* from France are reported in *red*, from Italy in *green* and from Spain in *blue*

In most cases, phenotypic records for multiple years were available (most commonly two or three years; only one for FW in BoxBo, as many as 6 for FW in PxF), collected between 1995 and 2013. The number of records spanned from 16 (TA, TxE, 2011) to 127 (FW, BC2, 2005). Figure 1 shows the boxplots of the phenotypic distributions per peach population and year of measurement. All phenotypes were approximately normally distributed. Descriptive statistics on the measured phenotypes per cross and year are reported in Additional file 1.

### Imputation of missing genotypes

After genotyping and editing for call-rate, residual missing genotypes were imputed using the localized haplotype clustering imputation (LHCI) method implemented in the software “Beagle” [27]. Originally developed for human genetics, LHCI has since found wide application also in animal and plant genetics (e.g. [11, 17, 20, 28]). Imputation was carried out in each cross separately, to avoid potential problems due to population heterogeneity.

The accuracy of imputation was measured. For each cross, a subset with no missing data was extracted from the total dataset, and increasing proportions of missing genotypes were then artificially introduced: 1, 2.5, 5, 7.5, 10, 12.5, 15, 17.5, 20, 22.5, 25, 27.5 and 30%. Missing genotypes were imputed using the LHCI method in Beagle. For each missing rate and cross, the imputation was repeated 10 times, each time resampling randomly the genotypes to be set to missing. The average proportion of correctly imputed genotypes over the 10 replicates for each missing rate was then used to estimate an empirical curve of the imputation accuracy in each peach cross.

### Assessment of the population structure

The population structure in the analysed peach population was assessed based on the kinship among all crosses. From imputed SNP genotypes, marker-based genomic relationships were estimated à la Astle & Balding [29]:

1 $$ \mathbf{G}=\frac{1}{L} \sum\limits_{l=1}^{L} \frac{\left(Z_{.,l} \right) \left(Z_{.,l} \right)'}{4p_{l} \left(1-p_{l} \right)}   $$

where *L* is the number of marker loci, *Z*_.,*l*_ is the *l*_*th*_ column of the matrix of marker genotypes corrected by allele frequencies, and *p*_*l*_ is the allele frequency at locus *l*.

From the kinship matrix in Eq. 1, the Neighbor-Joining (NJ) tree [30] of the 11 peach crosses was constructed.

### Repeatability model for genome-enabled predictions

For the prediction of fruit size, sugar content and acidity based on SNP genotypes, a GBLUP (Genomic Best Linear Unbiased Predictions) approach was used [31]. GBLUP was run for each peach population separately, and SNP with within-population MAF <1*%* were removed before the analysis. Since multiple measurements for the same trait were recorded in successive years on individual peach trees, a repeatability model was used to fit systematic, additive genetic and permanent environment effects [23]. In matrix notation, the model had the following form:

2 $$ \mathbf{y}=\mathbf{Xb}+\mathbf{Za}+\mathbf{Wpe}+\mathbf{e}   $$

where **y** is the vector of (repeated) observations for each of the three traits; **b** is the vector of fixed effects: the overall mean and the year of measurement (categorical); **a** is the vector of additive genetic effects; **p** **e** is the vector of permanent environment effects; **e** is the vector of residual effects; **X**, **Z** and **W** are incidence matrices which relate records in **y** to fixed, additive genetic and permanent environment effects, respectively. Residuals and permanent environment effects are assumed to be independent and normally distributed, with mean zero and variances $\mathbf {I}\sigma _{e}^{2}$ and $\mathbf {I}\sigma _{pe}^{2}$.The additive genetic effects are also assumed to follow a normal distribution, and have mean 0 and variance $\mathbf {G}\sigma _{a}^{2}$, where **G** is the matrix of genomic relationships –within cross– calculated as in Eq. 1. From variance components, the narrow sense heritability (*h* ^2^) and repeatability (*R*) were derived:

3 $$\begin{array}{@{}rcl@{}} h^{2}&=&\frac{\sigma_{a}^{2}}{\sigma_{a}^{2}+\sigma_{pe}^{2}+\sigma_{e}^{2}}  \end{array} $$

4 $$\begin{array}{@{}rcl@{}} R&=&\frac{\sigma_{a}^{2}+\sigma_{pe}^{2}}{\sigma_{a}^{2}+\sigma_{pe}^{2}+\sigma_{e}^{2}}  \end{array} $$

For specific combinations of peach cross and trait the sample size was rather small in terms of number of records and, especially, number of unique individuals (see Additional file 1). This, at times, made matrices singular and non-invertible, leading to numerical problems with the estimation of parameters. Therefore, the model in Eq. 2 was fit using either Restricted Maximum Likelihood (REML: [32]) or an MCMC approach [33].

### Estimation of the predictive ability

For each trait and cross, the predictive ability (*PA*) of model 2 was assessed through a 5-fold cross-validation. Plant records were randomly partitioned into 5 subsets of approximately equal size (from ∼12 records for TA in TxE, to ∼120 records for FW in PxF). In turn, the records in one subset were set to missing and predicted using the model trained in the remaining four subsets, until all subsets were once used as validation set and every observation was used both to train and validate the model. The 5-fold cross-validation was repeated 100 times, each time resampling different subsets, eventually yielding 500 replicates of the analysis (per peach cross, per trait). In each replicate, *h* ^2^, *R* and the predictive ability were calculated.

*PA* was calculated as the correlation between observed and predicted phenotype, $r(\hat {y},y)$, in the validation set. Predicted observations were obtained by summing up effects from the model (Eq. 2): $\hat {y}=\mu +\hat {year}+\hat {a}+\hat {pe}$. Estimates of *h* ^2^, *R* and $r(\hat {y},y)$ were averaged over the 500 replicates to obtain robust estimates of the central tendency and variability of the genetic parameters for fruit size and quality and of the accuracy of genomic predictions.

### Software used

Imputation of missing genotypes was performed using the *Beagle* software [27]. Variance components were estimated with a restricted maximum likelihood approach using the *Asreml* software [34] or with a MCMC approach using the BGLR R package [35]. Data manipulation, the parsing of results and plots were done using the *R* software [36].

## Results

### SNP genotypes and imputation accuracy

After imputation of residual missing genotypes, the proportion of monomorphic SNP ranged from 9.4*%* in T1E to 49.5*%* in MxR028, with an average of 35.8*%* over all crosses. After monomorphic SNPs were removed, the minor allele frequency (*MAF*) ranged from 21.20*%* (T1E) to 47.60*%* (BtxNr), with an average of 26.4*%* over all samples. Details on monomorphic markers frequency and MAF are reported in Table 1.

**Fig. 2:**
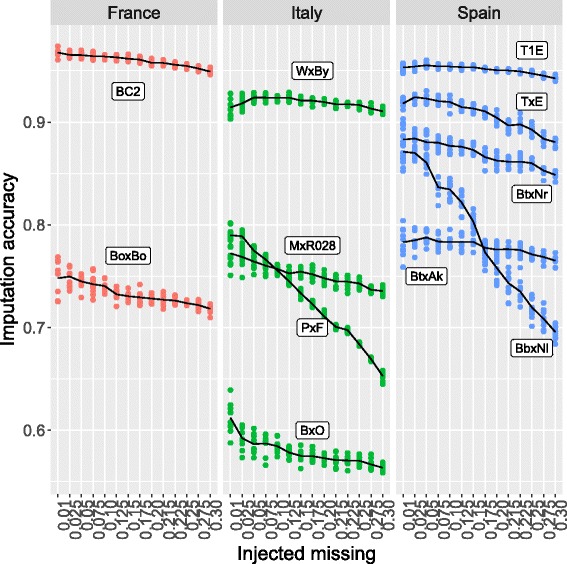
Imputation accuracy over increasing percentages of missing genotypes in the data. Results are from 10 replicates per cross (the line is the average). *Crosses* from France are reported in *red*, from Italy in *green* and from Spain in *blue*

The imputation accuracy was measured per cross as the ratio of correctly imputed genotypes over the total number of artificially introduced missing genotypes, for increasing missing rates (from 1 to 30%). Results from 10 repetitions (per cross, per missing rate) are reported in Fig. 2; the interpolating lines are the average accuracies. Standard deviations and further details can be found in Additional file 2.

The average imputation accuracy over all crosses and missing rates was 0.82, and varied from 0.96 (BC2) to 0.58 (BxO). Imputation accuracy was typically higher with low proportions of missing genotypes in the data: 0.97 in BC2 and 0.95 in T1E at 1% missing rate. The lowest imputation accuracy (0.564) was found for BxO at 30% missing genotypes. Most crosses showed a generally flat response to increasing missing rates, with imputation still performing well even with 20% or more missing genotypes. Exceptions were PxF and BbxNl, whose imputation accuracy dropped by 17 and 20 percentage points, respectively, between 1 and 30% missing rates.

### Kinship matrix and neighbor-joining tree

**Fig. 3:**
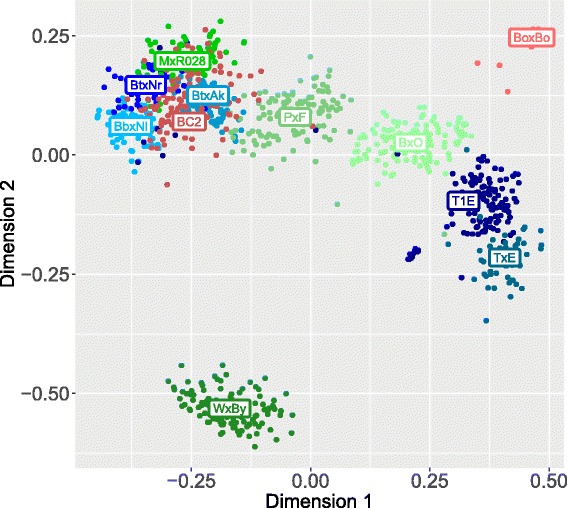
MDS plot of the matrix of genomic relationships across peach crosses: first two principal coordinates. *Crosses* from France are reported in shades of *red*, from Italy in shades of *green* and from Spain in shades of *blue*. Labels for each progeny are added for clarity

**Fig. 4:**
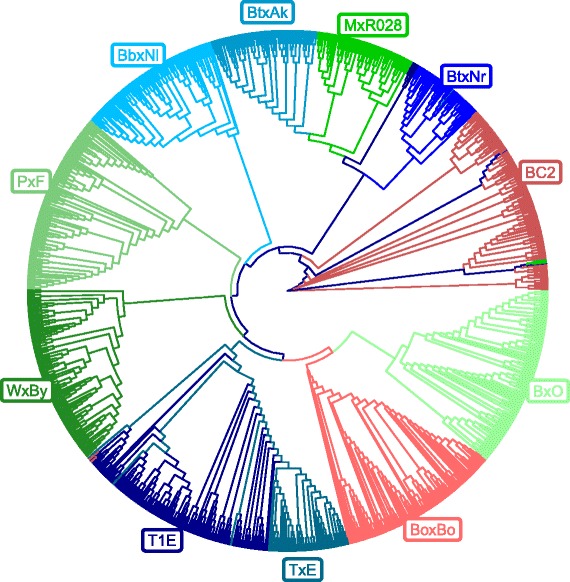
NJ tree of the 11 peach crosses analysed in this study. *Crosses* from France are reported in shades of *red*, from Italy in shades of *green* and from Spain in shades of *blue*. Labels for each progeny are added for clarity

From the multidimensional scaling of the reciprocal of the kinship matrix in Eq. 1 (1−*G*), principal coordinates and corresponding eigenvalues were obtained. The two first dimensions accounted for 27.5*%* of the genetic variability (50.3*%* the first five), and are plotted in Fig. 3: a strong population structure is apparent, with specific peach crosses clearly clustering together. BoxBo and WxBy clustered separately from other crosses: BoxBo formed a very compact cluster, while the WxBy cluster is more spread out. Five crosses (BbxNl, MxR028, BC2, BtxAk, BtxNr) formed an entangled cluster and higher-order dimensions are required to visually separate them. The highly structured nature of the dataset is confirmed by the Neighbor-Joining (NJ) tree [30] obtained from the across-population kinships and shown in Fig. 4.

### Heritabilities and repeatabilities

Average *h* ^2^ and *R*, with the corresponding variability, for the three phenotypes analysed are reported in Table 2. The average heritability for FW was mostly moderate, in the range from 0.207 (T1E) to 0.361 (TxE), except for crosses MxR028 (0.422), WxBy (0.468) and BxO (0.783), where high *h* ^2^ for FW were estimated. The standard deviation of estimated *h* ^2^ ranged from 0.034 (BC2) to 0.392 (TxE). For TA, average *h* ^2^ varied between 0.304 (PxF) and 0.832 (BbxNl), with standard deviation in the range 0.005 (PxF) - 0.261 (BC2). Average *h* ^2^ of SC ranged from 0.078 (BC2) to 0.861 (BbxNl) (std. dev.: 0.005 (PxF) - 0.269 (TxE)).

**Table 2:** Heritability (*h* ^2^), repeatability (*R*) and predictive ability (*PA*: $r(\hat {y},y)$) for fruit weight, sugar content and acidity in the 11 peach populations analysed in this study

Country	Cross	Trait	Avg(*h* ^2^)	Sd(*h* ^2^)	Avg(*R*)	Sd(*R*)	Avg(*PA*)	Sd(*PA*)
France	BoxBo	FW	0.22	0.22	0.34	0.28	0.42	0.39
France	BC2	TA	0.63	0.26	0.87	0.03	0.76	0.08
France	BC2	FW	0.24	0.03	0.25	0.03	0.54	0.06
France	BC2	SC	0.08	0.03	0.18	0.03	0.78	0.11
Italy	BxO	TA	0.34	0.07	0.44	0.04	0.52	0.09
Italy	BxO	FW	0.78	0.16	0.83	0.02	0.84	0.04
Italy	BxO	SC	0.63	0.14	0.70	0.03	0.69	0.06
Italy	PxF	TA	0.30	0.00	0.38	0.00	0.50	0.07
Italy	PxF	FW	0.33	0.15	0.45	0.16	0.49	0.24
Italy	PxF	SC	0.53	0.00	0.64	0.00	0.75	0.05
Italy	MxR028	TA	0.39	0.00	0.49	0.00	0.66	0.11
Italy	MxR028	FW	0.42	0.16	0.55	0.09	0.71	0.11
Italy	MxR028	SC	0.46	0.01	0.58	0.00	0.77	0.08
Italy	WxBy	FW	0.47	0.08	0.58	0.06	0.58	0.12
Spain	BbxNl	TA	0.83	0.02	0.83	0.02	0.83	0.04
Spain	BbxNl	FW	0.27	0.20	0.78	0.05	0.63	0.11
Spain	BbxNl	SC	0.86	0.04	0.89	0.03	0.67	0.09
Spain	BtxAk	TA	0.43	0.12	0.46	0.07	0.59	0.10
Spain	BtxAk	FW	0.29	0.21	0.57	0.10	0.70	0.17
Spain	BtxAk	SC	0.62	0.13	0.65	0.03	0.72	0.07
Spain	BtxNr	TA	0.50	0.08	0.65	0.04	0.60	0.11
Spain	BtxNr	FW	0.29	0.18	0.45	0.10	0.39	0.20
Spain	BtxNr	SC	0.57	0.11	0.71	0.04	0.68	0.13
Spain	T1E	TA	0.67	0.08	0.74	0.07	0.79	0.07
Spain	T1E	FW	0.21	0.20	0.54	0.20	0.53	0.29
Spain	T1E	SC	0.41	0.07	0.54	0.06	0.75	0.09
Spain	TxE	TA	0.67	0.13	0.78	0.08	0.58	0.19
Spain	TxE	FW	0.36	0.39	0.86	0.06	0.81	0.13
Spain	TxE	SC	0.21	0.27	0.79	0.08	0.66	0.16

**Fig. 5:**
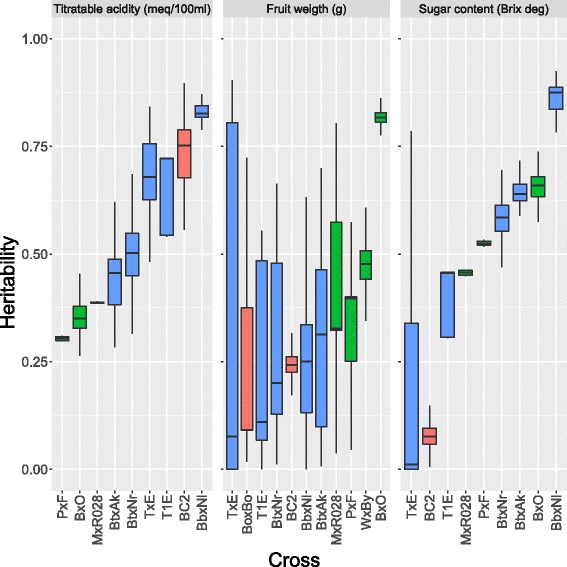
Boxplots of heritability estimates for acidity, fruit weight and sugar content in 11 peach populations. Results from 5-fold cross-validation repeated 100 times (500 replicates) are presented. *Crosses* from France are reported in *red*, from Italy in *green* and from Spain in *blue*. *Crosses* are ordered by increasing median value

**Fig. 6:**
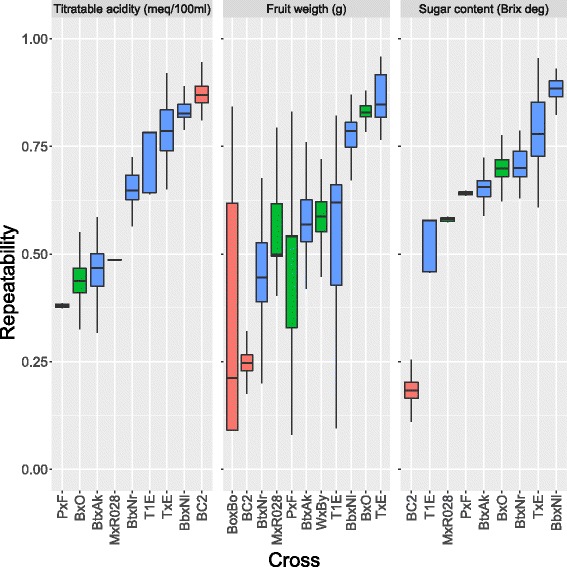
Boxplots of repeatability estimates for acidity, fruit weight and sugar content in 11 peach populations. Results from 5-fold cross-validation repeated 100 times (500 replicates) are presented. *Crosses* from France are reported in *red*, from Italy in *green* and from Spain in *blue*. *Crosses* are ordered by increasing median value

Repeatability estimates were on average 51.95*%* higher than corresponding *h* ^2^ estimates: from as little as 0.09*%* for TA in BbxNl, or 1.99*%* for FW in BC2, to as much as 273.9*%* (almost four times) for SC in TxE (from *h* ^2^=0.210 to *R*=0.787). The variability of repeatability estimates was substantially lower than that of heritability estimates (average coefficient of variation: 12.45*%* vs 36.05*%*). Figures 5 and 6 show the boxplots of the 500 *h* ^2^ and *R* estimates per trait and cross.

### Predictive ability

**Fig. 7:**
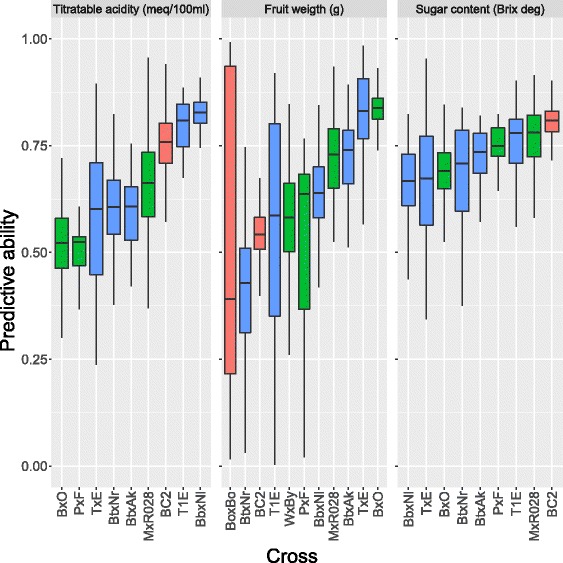
Boxplots of the estimated predictive ability for acidity, fruit weight and sugar content in 11 peach populations. Results from 5-fold cross-validation repeated 100 times (500 replicates) are presented. *Crosses* from France are reported in *red*, from Italy in *green* and from Spain in *blue*. *Crosses* are ordered by increasing median value

Predictive ability (PA, $r(\hat {y},y)$) was measured in the validation set from a 5-fold cross-validation scheme (Table 2). Each trial was repeated 100 times to assess the variability of *PA*. Figure 7 reports *PA* per trait and cross. Moderate values of predictive ability were observed when averaged over crosses: from 0.72 for SC, to 0.65 for TA and 0.60 for FW. When predicting TA the best average performance was achieved in BbxNl (0.83±0.044), the worst in PxF (0.5±0.074), with TxE and MxR028 showing high variability of results (standard deviation 0.19 and 0.11, respectively). For FW, the highest predictive ability was estimated in BxO (0.84±0.039) and the lowest in BtxNr (0.39±0.197), with BoxBo displaying by far the largest variability (standard deviation 0.395).

Finally, genomic predictions for SC showed the narrowest range, with average values all falling in the 0.66 (TxE) - 0.78 (BC2) interval. The most variable predictive ability was estimated in TxE (standard deviation 0.16).

## Discussion

The joint effort between this paper and the paper by Hernandez-Mora et al. [37] constitutes the first large work to investigate the applicability of genomics-assisted breeding for complex quantitative traits (QTL mapping and genome-enabled predictions) in *P. persica*. The genetic materials and phenotypic records in the two works largely overlap; however, Hernandez-Mora et al. focussed on QTL detection, and looked at genome-enabled predictions as a collateral result. In this study we provide further insights into genomic predictions in peach trees, considering also the variability of estimates in specific cross-trait combinations. We selected three quantitative continuous traits (fruit weight, sugar content, titratable acidity) based both on their commercial importance and on the availability of complete datasets spanning several crosses and years. The phenotypic information from records in different years has not been pooled, rather used to allow for the effect of permanent environment to be estimated. GBLUP was used for genomic predictions (and estimation of heritability and repeatability), instead of a weighted sum of QTL effects as in FlexQTL®;[38]. Additionally, we implemented a repetition protocol to ensure numerical stability in spite of the stochastic variability embedded in cross-validation and Gibbs samplings (BGLR). Finally, the accuracy of imputing missing genotypes in peach was measured in this work.

### General aspects: heritability, repeatability and estimation model

This study reports a systematic investigation of the applicability of genomic prediction models to key traits for peach fruit quality and marketability. Examples of genomic selection studies in fruit trees include apple [20, 39, 40], Japanese pear [21] and grapevine [41]. A comprehensive review is given by Iwata et al. [42].

Estimated heritability, repeatability and accuracy of genomic predictions varied widely across specific peach crosses and traits. Sample size and phenotypic variability are factors that can affect the absolute value and variability of estimated genetic parameters and genomic predictions. The average sample size (across years) varied dramatically: from 19.6 plants for TA in TxE to 237.5 plants for FW in WxBy. Substantial phenotypic variability was found: the phenotypic coefficient of variation ranged from 16.3 to 48.8*%* in FW, from 7.2 to 36.9*%* in SC, and from 10.4 to 73.1*%* in TA. This reflects the wide range of variability in the peach materials included in the study, and directly influences the estimates of *h* ^2^ and *R*. Additional files 3 and 4 show the coefficient of variation of estimated *h* ^2^, *R* and predictive ability as a function, respectively, of the average sample size and of the phenotypic coefficient of variation. It appears that for larger sample size and phenotypic variance, the estimates of parameters are more reliable (less variable), indicating that these two factors do affect the estimation of heritability, repeatability and the accuracy of genomic predictions.

Fruit phenotypes are affected by genetic, ontogenetic (age-related: i.e. consecutive years of growth) and environmental (i.e. climatic years) factors. These can be statistically separated to clarify their contribution to the observed phenotypes (e.g. [43, 44]). From a repeatability model, such as that in Eq. 2, the variance components due to the genetic, permanent and temporary environmental effects are estimated. The permanent environment actually catches the effect of consecutive years of growth, while the temporary environment captures the variability linked to the climatic conditions of specific years. For instance, for sugar content in MxR028, genetics, permanent and temporary environments account for, respectively, 45.6*%* (*h* ^2^), 12.4*%* (*R*−*h* ^2^) and 42% (1−*R*) of the phenotypic variability. Therefore, the repeatability model may be used as an alternative approach to estimating the genetic, ontogenetic and climatic effects in fruit trees. The less variable the estimates of the genetic parameters, the more reliable the approximations.

Compared to traditional QTL-oriented marker assisted selection, genomic selection is generally thought to perform better for selecting traits controlled by a large number of minor genes, each contributing a small proportion of the total phenotypic variability. The traits used in this study are largely polygenic (FW: [45] SC: [46]) and thus well suited for GBLUP and similar approaches (like SNP/RR-BLUP e.g. [47]), which build on the hypothesis of many small additive allele contributions to the phenotype (i.e. “infinitesimal model” [48]).

We therefore selected a GBLUP framework to apply a repeatability mixed model to the problem of estimating genetic parameters and genomic predictions for fruit weight and quality from SNP genotypes. The model in Eq. 2 was solved either through REML or MCMC, implemented, respectively, in a commercial (ASREML) and an open source (BGLR) software package. This nicely illustrates the difference between statistical model of analysis (GBLUP repeatability model), method of resolution, and specific algorithmic implementation into a software.

### Imputation accuracy

The imputation of missing genotypes has been repeatedly shown to be very accurate: e.g. 95% in humans [49], ∼99*%* in cattle [50], ∼98*%* in rice [28]. Lower imputation accuracy has been observed in other plant species: e.g. 84% in sugar beet [17], ∼80*%* in alfalfa [28]. Imputation errors may have a detrimental effect on the accuracy of genomic predictions (e.g. [51]), and there is therefore interest in assessing imputation accuracy when genotypes are used to predict phenotypes or breeding values. Our results showed that imputation accuracy is quite variable over peach crosses, with a difference of 35.5 percentage points between the best (BC2: 0.96) and the worst (BxO: 0.61) case. The response to increasing missing rates was quite flat in all crosses, with the exception of PxF and BbxNl. Such variable results suggest a strong influence of the genetic background of each population on genotype imputation in peach.

Putting together the average missing rates in the original datasets (Table 1) with the corresponding estimated imputation accuracies (Additional file 2), the amount of imputation errors in the data used for genomic predictions can be estimated in the range 0.88*%* (BbxNl) - 5.33*%* (TxE), with an average of 2.71*%*. Given the low initial missing rates, and the generally good imputation accuracy, there are therefore few residual imputation errors, which are expected to have negligible impact on genomic predictions.

### Population structure

Unaccounted population stratification is known to potentially have detrimental effects on genome-wide association studies [52–54] and genomic predictions [41, 55–57]: the association between SNP and phenotype may differ between (sub)populations or be in reverse phase. If possible, it may therefore be advisable, when analysing heterogeneous populations, to account for this in the model (e.g. [58, 59]).

In this study, peach crosses were analysed separately. Still, it is interesting to look at population structure, since this can help interpret the obtained results, and provide guidance for future modeling of genomic predictions in peach populations. The BoxBo resulted in a very compact and isolated cluster, and the relatively limited genetic variability may be related to the comparatively poorer accuracy of genomic predictions in this population. This cross is indeed a self pollination of a partially heterozygous variety. The WxBy cluster is more spread out and prediction accuracy was higher. The separation of this progeny from the other crosses is likely linked with the ornamental NJ Weeping parent —indeed ornamental germplasm is known to have undergone divergent breeding history compared to edible cultivated accessions [60, 61]. The separation of T1E and TxE from the main peach group of progenies from commercial peaches can be attributed to the origin of these populations from almond x peach crosses. The two crosses sharing a parent (T1E and TxE) clustered very closely together. Five crosses (BbxNl, MxR028, BC2, BtxAk, BtxNr) formed an entangled cluster and higher order dimensions are required to visually separate them.

### Accuracy of genomic predictions

The accuracy of genomic predictions for fruit weight, titratable acidity and sugar content was variable across and within crosses, but less so compared to estimates of heritability and repeatability: for FW, the average predictive ability (PA) ranged from 0.39 in BtxNr to 0.84 in BxO; for TA, it ranged between 0.50 in PxF and 0.83 in BbxNl; for SC average PA was in the range 0.66 (TxE) - 0.78 (BC2). The average standard deviation of PA was 0.17, 0.09 and 0.09 for FW, TA and SC respectively. Predictive abilities appear therefore to be more reliable and robust than estimates of variance components due to different sources of variation (additive genetic effects, permanent environment). This is related to the general observation that predictions and inference (e.g. trying to understand the relative influence of genetics and environment on the phenotype) are different problems, and a model may yield good predictions even when the underlying biological mechanisms are poorly understood or estimated (and vice-versa: [62]). In some cases, PA showed very low variability, like in BxO for FW or in BbxNl for TA; in other cases, PA was so variable to become practically unreliable, like FW in BoxBo, where predictive ability went from -0.5 to 0.99. The performance of genomic predictions can be influenced by the size of the analysed dataset, the heritability and repeatability of the trait, and by the phenotypic variability. Additional files 3 and 4 show the coefficient of variation of PA as a function of sample size and phenotypic variability (x-axis). In both figures, the general trend is that the variability of estimates tends to be larger with smaller sample size and smaller phenotypic variability. Additional file 5 shows the coefficient of variability of predictive ability vs the heritability. Again, the larger the heritability, the smaller the variability of predictions, hence their reliability.

Predictive ability is defined as the correlation between the observed phenotype and the phenotype predicted by the model, $r(\hat {y},y)$. In plant and animal breeding it is often of interest to predict not only the (future) phenotypic value of an individual, but also its unobserved breeding (genetic) value. By dividing predictive abilities by the square root of the heritability of the trait, the accuracy of genomic breeding values (GEBVs) can be estimated [63]:

5 $$ r_{g,\hat{g}}=\frac{r_{y,\hat{y}}}{\sqrt{h^{2}}}   $$

We thus obtained average GEBV accuracies of 0.82, 0.83 and 0.97 for fruit weight, acidity and sugar content, respectively. The higher GEBV accuracy for sugar content reflects the higher average predictive ability (0.72), and the lower variability of estimated heritability (average s.d. 0.088) and repeatability (average s.d. 0.034).

### Applications to management and breeding

Genomic selection is having a profound impact on plant breeding. Major drivers behind this success are the possibility of obtaining accurate genomic predictions even without pedigree data, a reference genome or dense marker genotypes, and the higher genetic gains per unit of time that are likely to be achieved (2-3 fold: [64–66]). This is especially true for plant species with a long breeding cycle, since selection candidates can be chosen at a much earlier stage than in traditional breeding programmes. Fruit trees are characterised by a long juvenile phase; in peach breeding programmes, the average generation interval spans 5–7 years, taking into account the length of the juvenile period and repeated years of phenotypic evaluation, and thus the benefits from shortened cycles through genome-enabled predictions are evident.

The benefits of genomic selection stem not only from accurate genome-enabled predictions and shorter generation intervals, but also from potentially lower phenotyping costs. Collecting phenotypes in fruit trees is costly [67], and restricting phenotypic evaluation to smaller subsets of progenies for shorter times may be beneficial. Coupling genomics, high-throughput phenotyping [68, 69], databases and tools for breeding has the potential of creating effective platforms for genomics-assisted breeding in all plant species (e.g. rice, [70]), particularly in fruit trees, including peach (see Iwata et al. 2016 for a review [42]).

Genome-enabled predictions are mainly used to select breeding candidates in genetic improvement schemes. However, in species with a long life-cycle, accurate genome-enabled predictions may be particularly useful to predict future phenotypes of the plant: e.g. which plants are most likely to repeatedly give a certain production in successive years, which plants to cull, keep, fertilize, which plants are expected to be more resilient to temporary environmental effects (e.g. climatic variations). Additionally, in general breeders/farmers tend to prefer “repeatable/reliable” plants which show little variability in phenotypes from year to year. Resende et al. [71] showed that genomic predictions modelled at early age did not appear to perform well in predicting phenotypes at later ages (6 years). In our study, we modeled multiple records over successive years and obtained accurate genome-enabled predictions of phenotypes in peach. This indicates that there may be value from the application of repeatability GBLUP models in peach breeding.

## Conclusions

In this paper, results from a repeatability GBLUP model for fruit weight, sugar content and titratable acidity in peach trees were reported. This is the first work to show the applicability of genomic predictions in *P. persica*. A very diverse set of peach crosses was used, in terms of genetic background of the germplasm, phenotypic variability and, especially, sample size. Still, the obtained results are very promising for the application of genomic selection to peach breeding programmes. The accuracy of imputing missing genotypes was as high as 96%, and the genomic predictive ability was on average 0.65, but could be as high as 0.84 for fruit weight or 0.83 for titratable acidity. The estimated repeatability may prove very useful in the management of the typical long cycles involved in peach productions, since it may indicate which plants bear the potential of being more resilient to temporary fluctuations and give repeatable performances. Additionally, the repeatability model may prove valuable in disentangling genetic, ontogenetic and environmental effects in the analysis of complex traits.

All together, the results of this work suggest that the implementation of genomic selection may be very advantageous in *P. persica*, for it can realistically lead to higher genetic gains per unit of time, improved management of the orchard and reduced costs of breeding programs.

## Additional files

Additional file 1 Detailed phenotypic data. Phenotypic data per year, cross and trait, comprising descriptive statistics. (CSV 3 kb)

Additional file 2 Imputation accuracy. Statistics on imputation accuracy of missing genotypes, per cross and injected missing levels. (CSV 5 kb)

Additional file 3 Effect of sample size. Figure reporting, for each trait, the coefficient of variation of heritability, predictive ability, and repeatability as functions of sample size. (PDF 6 kb)

Additional file 4 Effect of phenotypic variability. Figure reporting, for each trait, the coefficient of variation of heritability, predictive ability, and repeatability as functions of the coefficient of variation of each phenotipic trait. (PDF 6 kb)

Additional file 5 Coefficient of variation of the predictive ability vs heritability. Figure reporting, for each trait, the coefficient of variation of predictive ability as function of the heritability of the trait in each progeny. Fruit weight in red, Sugar content in green and Titratable acidity in blue. (PDF 5 kb)

## Abbreviations

FW: Fruit weight
GBLUP: Genomic best linear unbiased predictions
GEBV: Genomic estimated breeding value
GS: Genomics selection
MAF: Minor allele frequency
PA: Predictive ability
SC: Sugar content
SNP: Single nucleotide polymorphism
TA: Titratable acidity
