# Supplementary figures and images for: Genome-enabled predictions for fruit weight and quality from repeated records in European peach progenies
Source: BMC Genomics. 2017 Jun 6;18:432. doi: 10.1186/s12864-017-3781-8 (PMC5460546; doi:10.1186/s12864-017-3781-8)

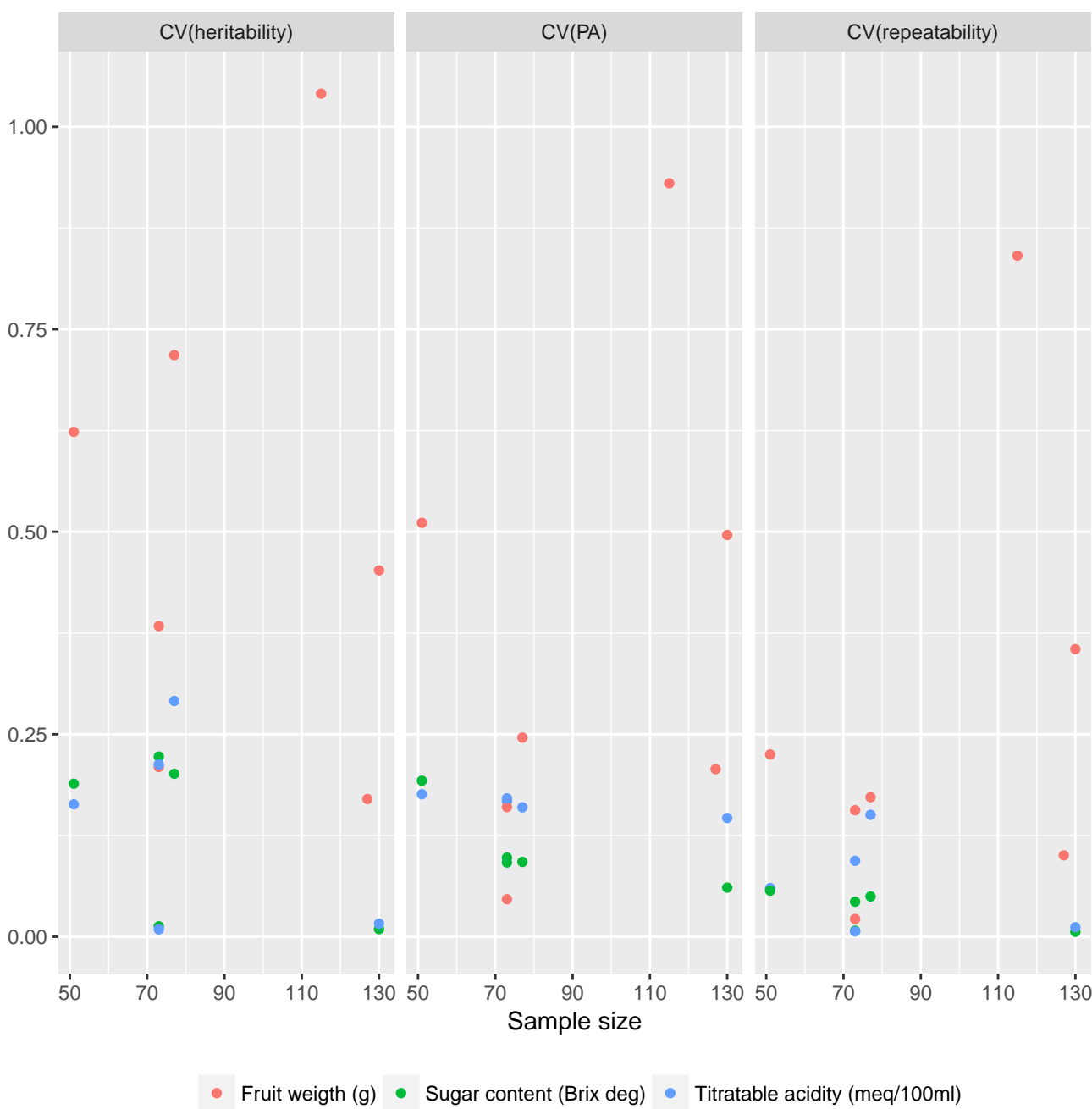

Supplement: Supplementary file 3 — Effect of sample size. Figure reporting, for each trait, the coefficient of variation of heritability, predictive ability, and repeatability as functions of sample size. (PDF 6 kb) [file 12864_2017_3781_MOESM3_ESM.pdf]

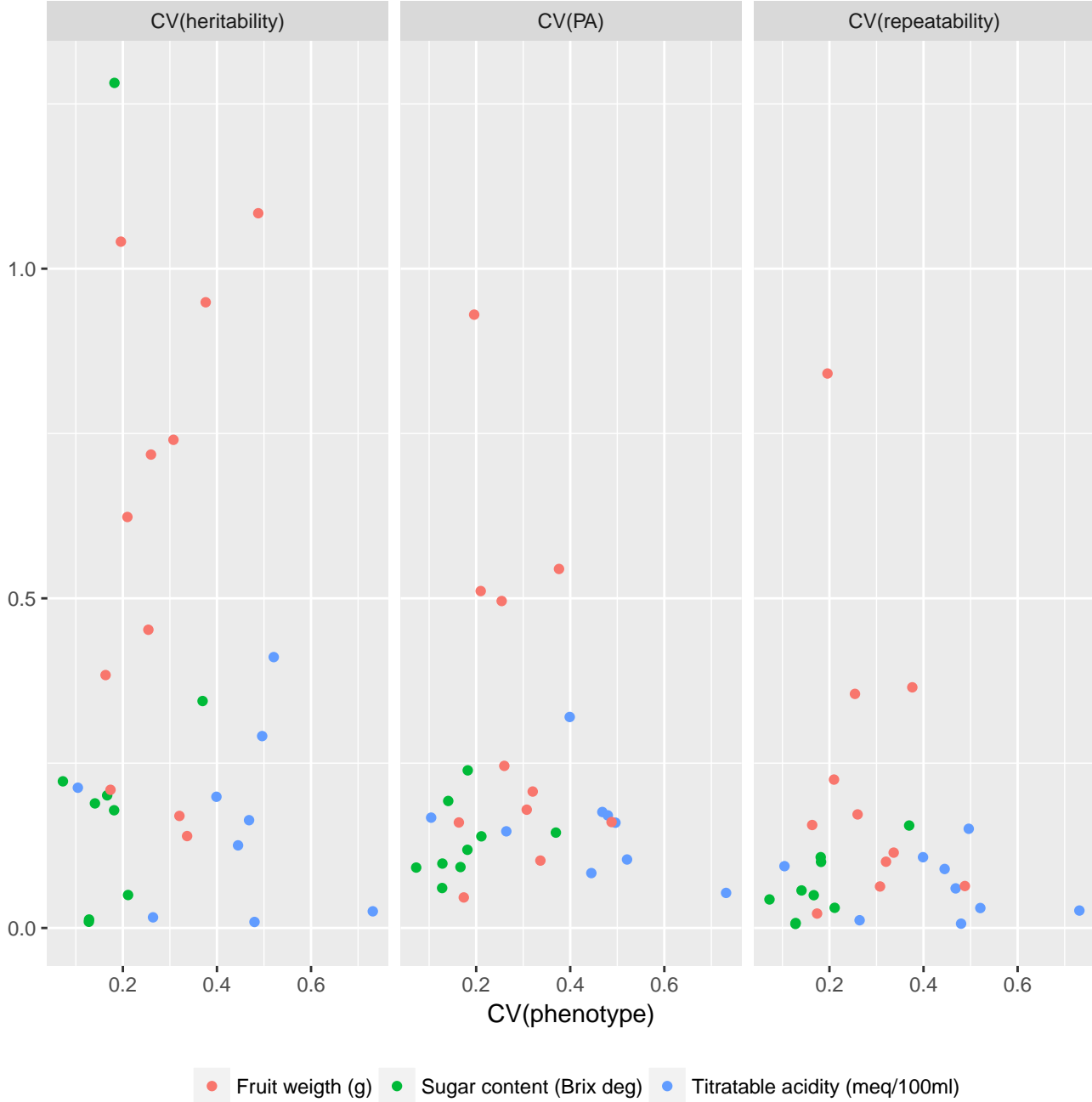

Supplement: Supplementary file 4 — Effect of phenotypic variability. Figure reporting, for each trait, the coefficient of variation of heritability, predictive ability, and repeatability as functions of the coefficient of variation of each phenotipic trait. (PDF 6 kb) [file 12864_2017_3781_MOESM4_ESM.pdf]

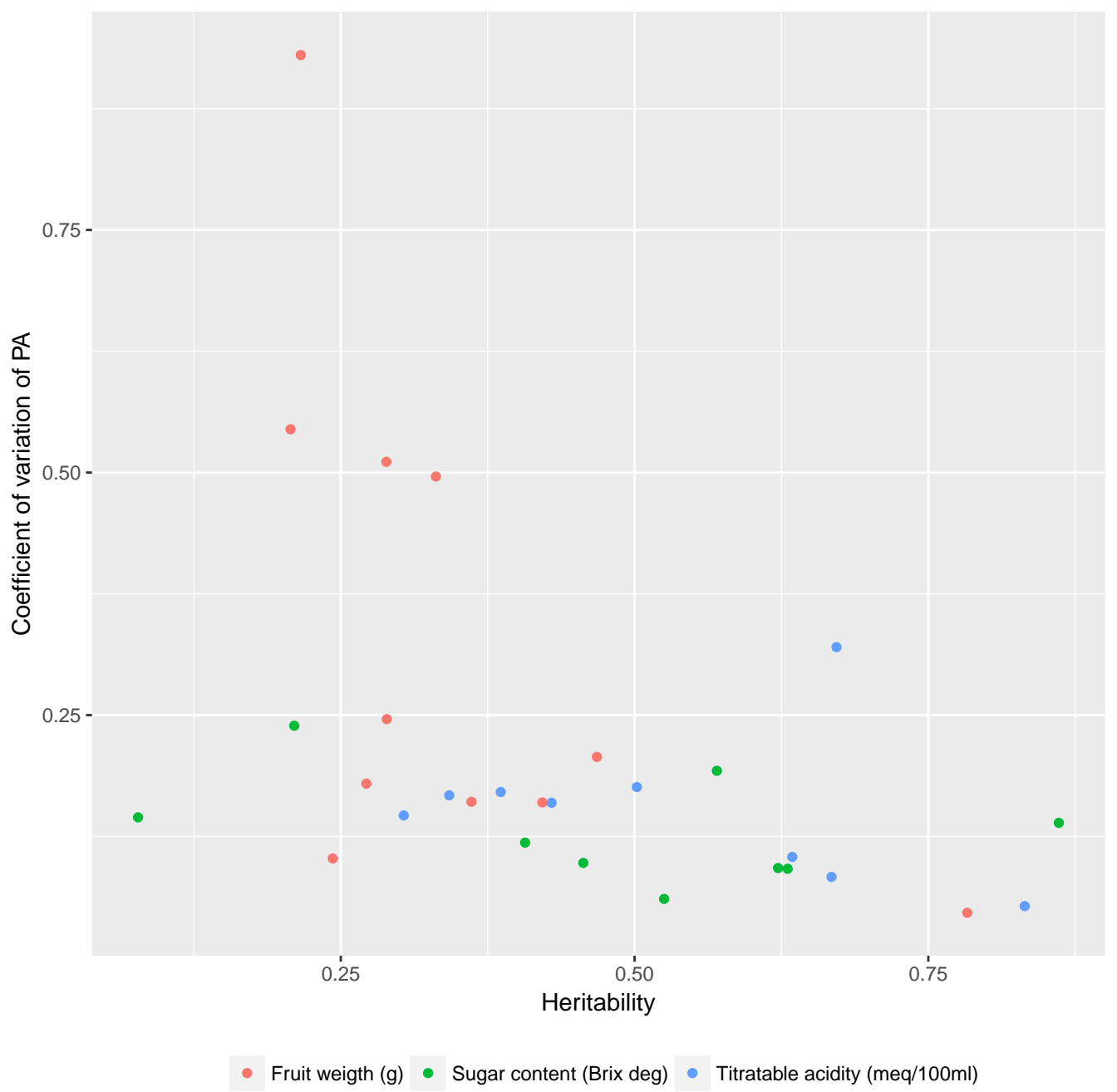

Supplement: Supplementary file 5 — Coefficient of variation of the predictive ability vs heritability. Figure reporting, for each trait, the coefficient of variation of predictive ability as function of the heritability of the trait in each progeny. Fruit weight in red, Sugar content in green and Titratable acidity in blue. (PDF 5 kb) [file 12864_2017_3781_MOESM5_ESM.pdf]
